# Supplementary figures and images for: Glycation of Whey Proteins Increases the Ex Vivo Immune Response of Lymphocytes Sensitized to β-Lactoglobulin
Source: Nutrients. 2023 Jul 12;15(14):3110. doi: 10.3390/nu15143110 (PMC10384914; doi:10.3390/nu15143110)

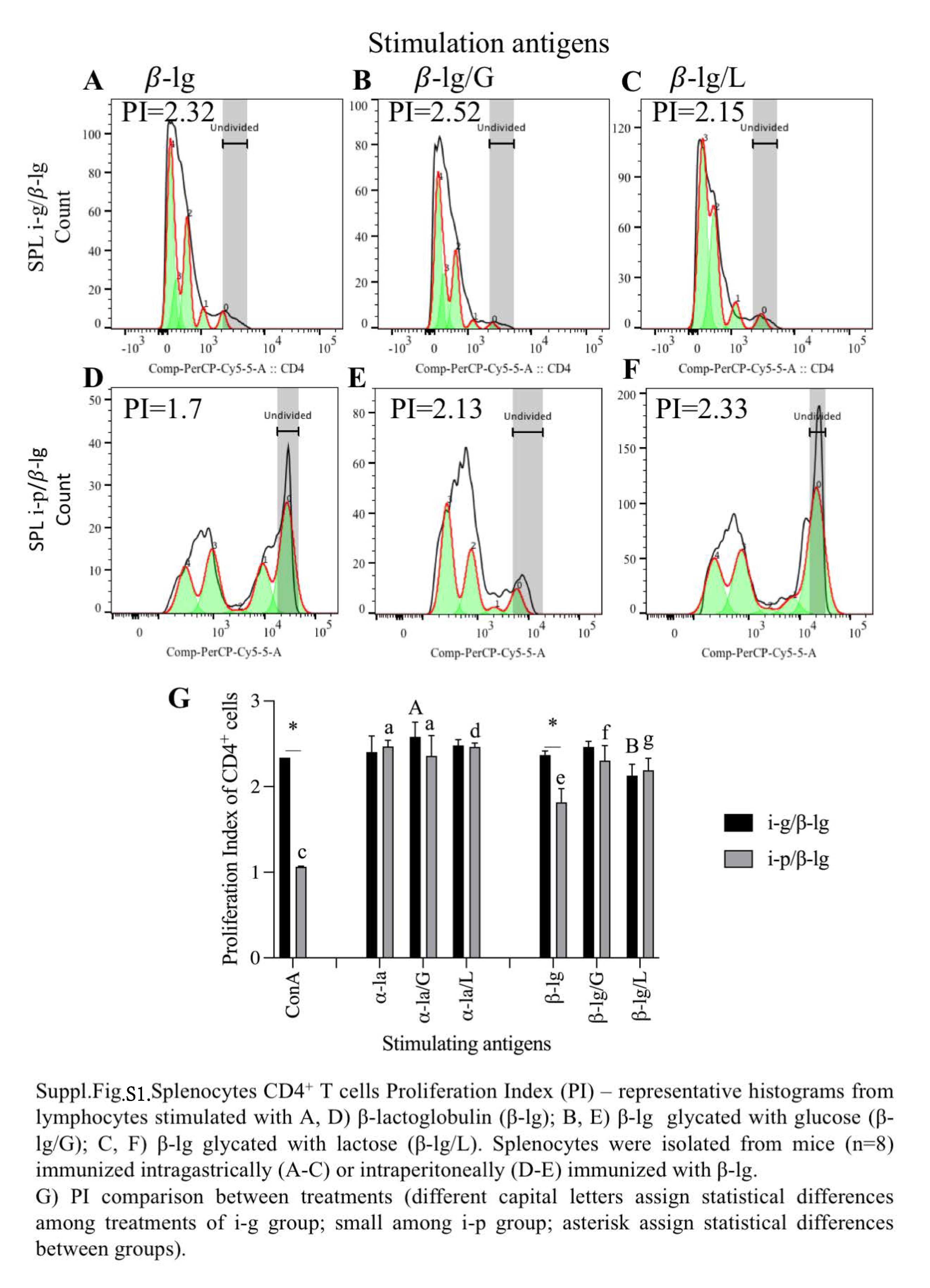

Supplement: Supplementary file 1 [file nutrients-15-03110-s001.zip › nutrients-2443502-supplementary.tif]
